# A Ribonucleoprotein Complex Protects the Interleukin-6 mRNA from Degradation by Distinct Herpesviral Endonucleases

**DOI:** 10.1371/journal.ppat.1004899

**Published:** 2015-05-12

**Authors:** Mandy Muller, Stephanie Hutin, Oliver Marigold, Kathy H. Li, Al Burlingame, Britt A. Glaunsinger

**Affiliations:** 1 Department of Plant and Microbial Biology, University of California, Berkeley, Berkeley, California, United States of America; 2 Department of Pharmaceutical Chemistry, University of California, San Francisco, San Francisco, California, United States of America; 3 Department of Cell and Molecular Biology, University of California, Berkeley, Berkeley, California, United States of America; Southwestern Medical Center, UNITED STATES

## Abstract

During lytic Kaposi’s sarcoma-associated herpesvirus (KSHV) infection, the viral endonuclease SOX promotes widespread degradation of cytoplasmic messenger RNA (mRNA). However, select mRNAs escape SOX-induced cleavage and remain robustly expressed. Prominent among these is interleukin-6 (IL-6), a growth factor important for survival of KSHV infected B cells. IL-6 escape is notable because it contains a sequence within its 3’ untranslated region (UTR) that can confer protection when transferred to a SOX-targeted mRNA, and thus overrides the endonuclease targeting mechanism. Here, we pursued how this protective RNA element functions to maintain mRNA stability. Using affinity purification and mass spectrometry, we identified a set of proteins that associate specifically with the protective element. Although multiple proteins contributed to the escape mechanism, depletion of nucleolin (NCL) most severely impacted protection. NCL was re-localized out of the nucleolus during lytic KSHV infection, and its presence in the cytoplasm was required for protection. After loading onto the IL-6 3’ UTR, NCL differentially bound to the translation initiation factor eIF4H. Disrupting this interaction, or depleting eIF4H, reinstated SOX targeting of the RNA, suggesting that interactions between proteins bound to distant regions of the mRNA are important for escape. Finally, we found that the IL-6 3’ UTR was also protected against mRNA degradation by the vhs endonuclease encoded by herpes simplex virus, despite the fact that its mechanism of mRNA targeting is distinct from SOX. These findings highlight how a multitude of RNA-protein interactions can impact endonuclease targeting, and identify new features underlying the regulation of the IL-6 mRNA.

## Author Summary

During replication of Kaposi’s sarcoma-associated herpesvirus (KSHV), the vast majority of mRNAs in the cytoplasm are cleaved and degraded by the viral nuclease SOX. However, some mRNAs escape this fate, including the transcript encoding the immunoregulatory cytokine IL-6. Here, we discover that this escape is mediated by a group of proteins that associates with a sequence element on the IL-6 mRNA. One of these proteins is nucleolin (NCL), a factor with diverse roles in RNA processing that is frequently co-opted during viral infection. During KSHV replication, a proportion of NCL is redirected from the nucleolar subcompartment of the nucleus into the cytoplasm, where it binds both the IL-6 3’ UTR and a complex of cellular proteins including the translation initiation factor eIF4H. This network of interactions is required for escape from virus-induced degradation. Collectively, these findings reveal novel interplay between the SOX escapees and the cellular mRNA stabilization machinery, and shed light on the complex crosstalk between viruses and hosts over the control of gene expression.

## Introduction

The posttranscriptional fate of mRNA, including translation, subcellular localization, and stability, is tightly controlled through complex networks of RNA-protein interactions. Many mRNA regulatory elements are located in the 3’ untranslated region (UTR), where they recruit factors that control the levels of the mRNA and its encoded protein both during homeostasis and in response to changes in the cellular environment [1]. In many cases the mechanisms by which these RNA-protein complexes assemble to direct a particular outcome remain unknown, although the best characterized elements are those that promote rapid degradation of mRNAs through recruitment of specific decay enzymes [2–4]. In this regard, mRNA stability is a key point of regulation that is readily engaged during pathogenesis.

Viruses have evolved ways to both circumvent and hijack cellular mRNA decay pathways [5,6]. In particular, gamma-herpesviruses (HVs), including Kaposi’s sarcoma-associated herpesvirus (KSHV) and Epstein-Barr virus (EBV), use RNA degradation as a means to broadly control both cellular and viral gene expression [7–10]. During their lytic replication cycle, gamma-HVs promote widespread acceleration of mRNA decay through the activity of the virally-encoded mRNA-specific endonuclease SOX. SOX internally cleaves cytoplasmic mRNAs in a site-specific manner and promotes their subsequent degradation by the cellular 5’-3’ exonuclease Xrn1 [11]. The importance of SOX-induced mRNA degradation has been demonstrated *in vivo* using the model virus murine gamma-HV 68 (MHV68), which displays defects in viral trafficking, cell type specific replication, and latency establishment upon introduction of a point mutation in SOX that selectively inhibits its mRNA degradation activity [7,9].

Despite this widespread mRNA degradation, approximately one-third of mRNAs appear to escape SOX-induced cleavage. Although in many instances ‘escape’ is likely a reflection of a secondary transcriptional compensation rather than a failure of SOX to cleave the mRNA, a subset of escapees are truly refractory to SOX targeting [12,13]. These are thought to escape SOX cleavage either because they lack a functional SOX targeting sequence or because they possess specific protective features that render them inaccessible to the viral nuclease. This latter class of escapees is of particular interest, as their characterization could reveal pathways of mRNA regulation that are inaccessible to viral or cellular endonucleases.

Interleukin-6 (IL-6) is an immunomodulatory cytokine important for survival of KSHV-infected B cells [14–16], and its mRNA is directly refractory to SOX-induced decay [17,18]. IL-6 expression spikes during KSHV infection both as a consequence of transcriptional and post-transcriptional control by the virus [17,18]. The ability of the IL-6 mRNA to escape SOX cleavage has been mapped to a specific protective sequence that resides within its 3’ UTR [18]. Fusion of the IL-6 3’ UTR to an mRNA that is normally targeted by SOX renders the mRNA protected, indicating that this RNA element somehow overrides the SOX targeting mechanism. This element recruits a largely undefined complex of cellular proteins, although two components have been identified as HuR and AUF1 and shown to participate in the protective phenotype [18].

Here, we sought to gain a more detailed understanding of how the IL-6 3’ UTR promotes escape from viral endonuclease targeting. Using a ribonucleoprotein (RNP) purification strategy coupled with mass spectrometry, we identified a set of proteins that specifically associate with this protective sequence. Depletion of at least five of these proteins adversely impacts protection, suggesting that the complex as a whole impacts SOX targeting. Among these, nucleolin (NCL) emerged as having the most robust contribution to IL-6 mRNA escape. We found that its re-localization during infection, coupled with specific long-range protein interactions formed only in the context of RNA binding, are prominent components of the protective phenotype. Finally, we demonstrate that the IL-6 3’ UTR also blocks mRNA degradation by the unrelated herpes simplex virus endonuclease vhs, suggesting a protective mechanism that operates across distinct endonuclease targeting strategies.

## Results

### Identification of the ribonucleoprotein complex associated with SOX resistance element

**Fig 1:**
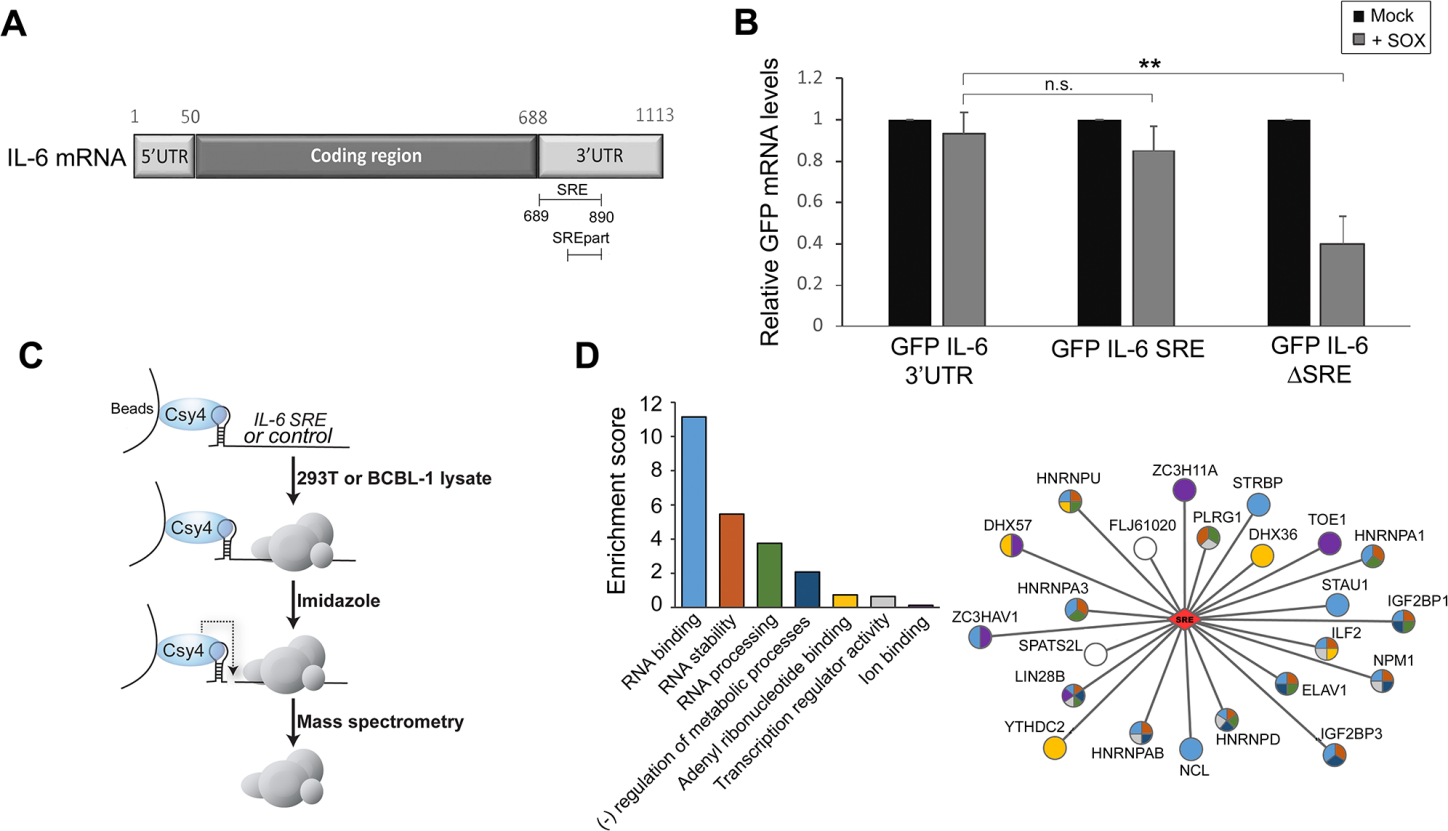
Identification of the RNP complex associated with the IL-6 SRE. **(A)** Schematic representation of the IL-6 mRNA. SRE refers to the complete protective sequence, whereas SRE_part_ refers to the portion of the SRE characterized previously [18]. **(B)** 293T cells were transfected with the indicated GFP-IL-6 fusion constructs in the presence or absence of a plasmid expressing SOX. After 24 h, total RNA was harvested and subjected RT-qPCR to measure GFP levels. Statistical significances here and for the following figures were determined by Student *t* test (* p<0.1; ** p<0.05; *** p<0.01) **(C)** Schematic representation of the Csy4-based RNP purification strategy. **(D)** A GO term analysis was performed using DAVID bioinformatics database on the high confidence proteins identified by Mass Spectrometry. The histogram on the left shows enrichment scores for the 7 clusters found in the GO term analysis. The network representation on the right shows the 23 proteins identified by MS as SRE binding proteins with the colors in the nodes showing their GO term association based on DAVID clustering.

The majority of cellular mRNAs, as well as reporter mRNAs such as GFP, are endonucleolytically cleaved by the KSHV SOX protein and subsequently degraded. However, the 3’ UTR of the IL-6 mRNA contains a sequence element that protects it against SOX cleavage [18]. Fusion of the IL-6 3’ UTR to a GFP reporter mRNA (GFP-3’IL-6) prevents SOX-induced cleavage, indicating that protection is transferrable. A 100 nucleotide (nt) region of the IL-6 3’ UTR (nt 790–890) is known to be involved in protection [18]. However, deletion of this 100 nt sequence does not eliminate protection from SOX, suggesting that additional flanking sequences might also contribute to escape. To more precisely define the region involved in the escape mechanism, we deleted larger fragments in IL-6 3’UTR, and identified a 200 nt-long sequence encompassing the original element (nt 689–890) that was both necessary and sufficient to confer resistance of the GFP-3’IL-6 fusion to cleavage by SOX (**Fig 1A**). We refer to this domain as the SOX-resistant element (SRE). RT-qPCR measurements of GFP mRNA levels showed that deletion of the SRE (GFP-IL-6SRE) eliminated protection from SOX-induced decay, whereas fusion of just the 200 nt SRE to GFP (GFP-IL-6 SRE) was sufficient to confer protection against SOX in transfected 293T cells (**Fig 1B**). These results were confirmed by measuring the half-life of GFP 3’ IL-6, SRE and ΔSRE in the presence or absence of SOX (**S1 Fig**). As observed previously, removing the SRE from the reporter results in stabilization of the transcript, due to the deletion of portions of AU-rich destabilization elements present in the IL-6 3’ UTR [18].

Sequence elements that impact mRNA stability generally function through the specific recruitment of RNA binding proteins that control message fate. To identify the set of factors specifically associated with the SRE, we applied a recently developed ribonucleoprotein (RNP) purification tool based on the conditional activity of the Csy4 ribonuclease from the bacterial CRISPR antiviral system (**Fig 1C**) [19]. Briefly, the Csy4 variant H29A/S50C binds extremely tightly (50 pm KD) to a 28 nt CRISPR RNA hairpin, and can be activated to cleave at a precise position in the hairpin in the presence of imidazole [19]. A hairpin-fused RNA segment and its associated RNP complex can therefore be purified by incubation over beads bound by recombinant Csy4 H29A/S50C, released in the presence of imidazole, and subjected to mass spectrometry (MS) to identify each of the bound proteins.

The CRISPR hairpin sequence was thus fused to the IL-6 SRE or, as a control, to an unrelated sequence corresponding to IL-6 coding region similar in size (nt 251 to 450), and the *in vitro* transcribed RNAs were bound to Csy4-coupled beads. Each fragment was then incubated with lysates from B cells stably infected with KSHV (TREX-BCBL-1) or from 293T cells, as the latter were used for the initial IL-6 SRE characterization experiments and thus contain the necessary cohort of factors required for SRE-mediated protection. After stringent washing, the RNP complexes were released by imidazole treatment and subjected to MS (**Fig 1C**). Of the 450 proteins identified by MS (**S1 Table**), 23 were specifically associated with or strongly enriched (at least 7-fold over the control) on the SRE-containing IL-6 RNA from both TREX-BCBL-1 and 293T lysates (**Table 1**). Each of these 23 proteins had a minimum of 3 peptide hits from the IL-6 SRE RNA purification, and a maximum of 1 peptide hit from the control RNA purification. Both AUF1 and HuR, the two known components of the SRE RNP [18], were re-identified as specific SRE-binding proteins in this manner, indicating that this is a robust methodology for revealing functionally relevant RNA-protein interactions. GO term analysis of this set of SRE-binding proteins revealed seven functional groupings, with a clear enrichment of RNA binding proteins and proteins involved in RNA regulation, as would be expected for factors that control the post-transcriptional fate of an mRNA (**Fig 1D** and **S2 Table**).

**Table 1:** SRE binding proteins.

				BCBL		293T	
				*IL-6 SRE*	*IL-6 control*	*IL-6 SRE*	*IL-6 control*
Official gene symbol	Uniprot accession number		Description	# of peptides	# of peptides	# of peptides	# of peptides
NCL*	P19338		Nucleolin	25	1	22	0
DHX36*	Q9H2U1		DEAH (Asp-Glu-Ala-His) box polypeptide 36	8	0	13	0
IGF2BP1*	Q9NZI8		insulin-like growth factor 2 mRNA binding protein 1	5	0	12	0
HNRNPAB*	Q53F64		heterogeneous nuclear ribonucleoprotein A/B	7	0	9	0
HNRNPU*	Q00839		heterogeneous nuclear ribonucleoprotein U	5	1	10	0
HNRNPD **	Q12771		heterogeneous nuclear ribonucleoprotein D—also known as AUF1	8	0	6	1
YTHDC2*	Q9H6S0		YTH domain containing 2	4	0	10	0
NPM1*	P06748		Nucleophosmin	7	0	6	0
DHX57*	Q6P158		DEAH (Asp-Glu-Ala-Asp/His) box polypeptide 57	5	0	8	0
ZC3HAV1*	Q7Z2W4		Zinc finger CCCH-type antiviral 1	10	0	3	0
**ILF2**	Q12905		Interleukin enhancer-binding factor 2	5	0	7	0
**HNRNPA1**	P09651		Heterogeneous nuclear ribonucleoprotein A1	5	1	5	0
**IGF2BP3**	O00425		Insulin-like growth factor 2 mRNA-binding protein 3	6	0	4	0
STAU1*	O95793		Staufen double-stranded RNA-binding protein 1	3	0	7	0
ELAVL1 **	Q15717		ELAV-like RNA binding protein 1—also known as HuR	4	0	5	1
**LIN28B**	Q6ZN17		Lin-28 homolog B	5	0	4	0
**TOE1**	Q96GM8		Target of EGR1 member 1	4	0	5	0
**FLJ61020**	B4E0W4		cDNA FLJ61020, highly similar to Heterogeneous nuclear ribonucleoprotein D0	4	0	4	1
**ZC3H11A**	O75152		Zinc finger CCCH-type containing 11A	5	0	3	0
**SPATS2L**	Q9NUQ6		spermatogenesis associated, serine-rich 2-like	7	0	1	0
**PLRG1**	O43660		Pleiotropic regulator 1	3	0	4	0
**HNRNPA3**	P51991		Heterogeneous nuclear ribonucleoprotein A3	4	0	3	0
**STRBP**	Q96SI9		Spermatid perinuclear RNA-binding protein	3	0	4	0

* proteins selected in this study for siRNA assay

** proteins previously identified as involved in IL-6 escape from SOX degradation [18]

### Multiple SRE-binding proteins contribute to protection from SOX-induced degradation

**Fig 2:**
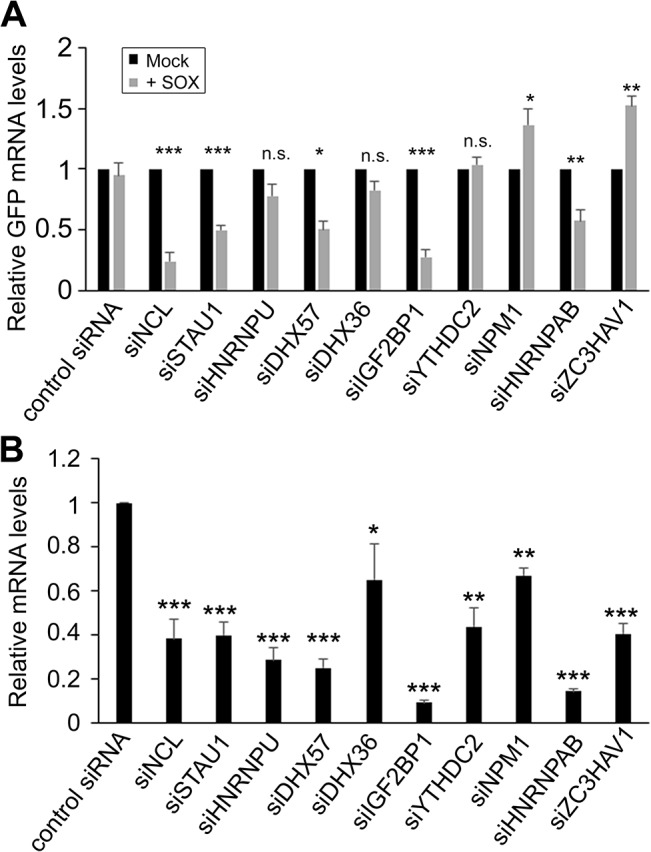
SRE-binding proteins contribute to protection from SOX-mediated decay. **(A)** 293T cells were transfected with siRNAs targeting each of indicated SRE binding proteins. 48h post-siRNA transfection, cells were further transfected with GFP-3’ IL-6 plasmid in the presence or absence of SOX. Cells were harvested 24h later and subjected to RT-qPCR to measure GFP and 18S levels. **(B)** Efficiency of siRNA-mediated depletion of the indicated SRE binding proteins was assessed by RT-qPCR.

To determine whether the complex of SRE-binding proteins was involved in the IL-6 escape mechanism, we selected 10 candidates for further analysis based on the robustness of their interaction and their putative or characterized roles in the regulation of RNA stability. These included nucleolin (NCL; the interaction with the most peptide hits), as well as STAU1, hnRNP U, DHX57, and DHX36, IGF2BP1, YTHDC2, NPM1, HNRNPAB and ZC3HAV1. Each factor was individually depleted from 293T cells using specific siRNAs, and the abundance of the GFP-3’IL-6 mRNA in the presence and absence of SOX was measured by RT-qPCR (**Fig 2A and 2B**). The SRE-containing GFP-3’IL-6 mRNA was protected against SOX-induced degradation in 293T cells transfected with a control nonspecific siRNA (**Fig 2A**). However, siRNA-mediated depletion of five out of the ten SRE binding proteins significantly decreased the protective effect of the IL-6 3’ UTR (**Fig 2A**). Out of these five proteins, only NCL knock down resulted in a reduced steady state level of the reporter independently of SOX (**S2 Fig**), which is not surprising given its known role as a regulator of RNA maturation [20].

It is possible that the effect of these SRE binding protein on IL-6 escape are underestimations of the contribution of each factor towards SRE-mediated protection, as the siRNA treatments resulted in only partial depletion of each endogenous transcript (**Fig 2B**). However, these data indicate that at least a subset of the SRE-binding proteins we identified by MS are functionally linked to IL-6 escape from degradation by SOX.

### The nucleolin-SRE interaction is required for escape

**Fig 3:**
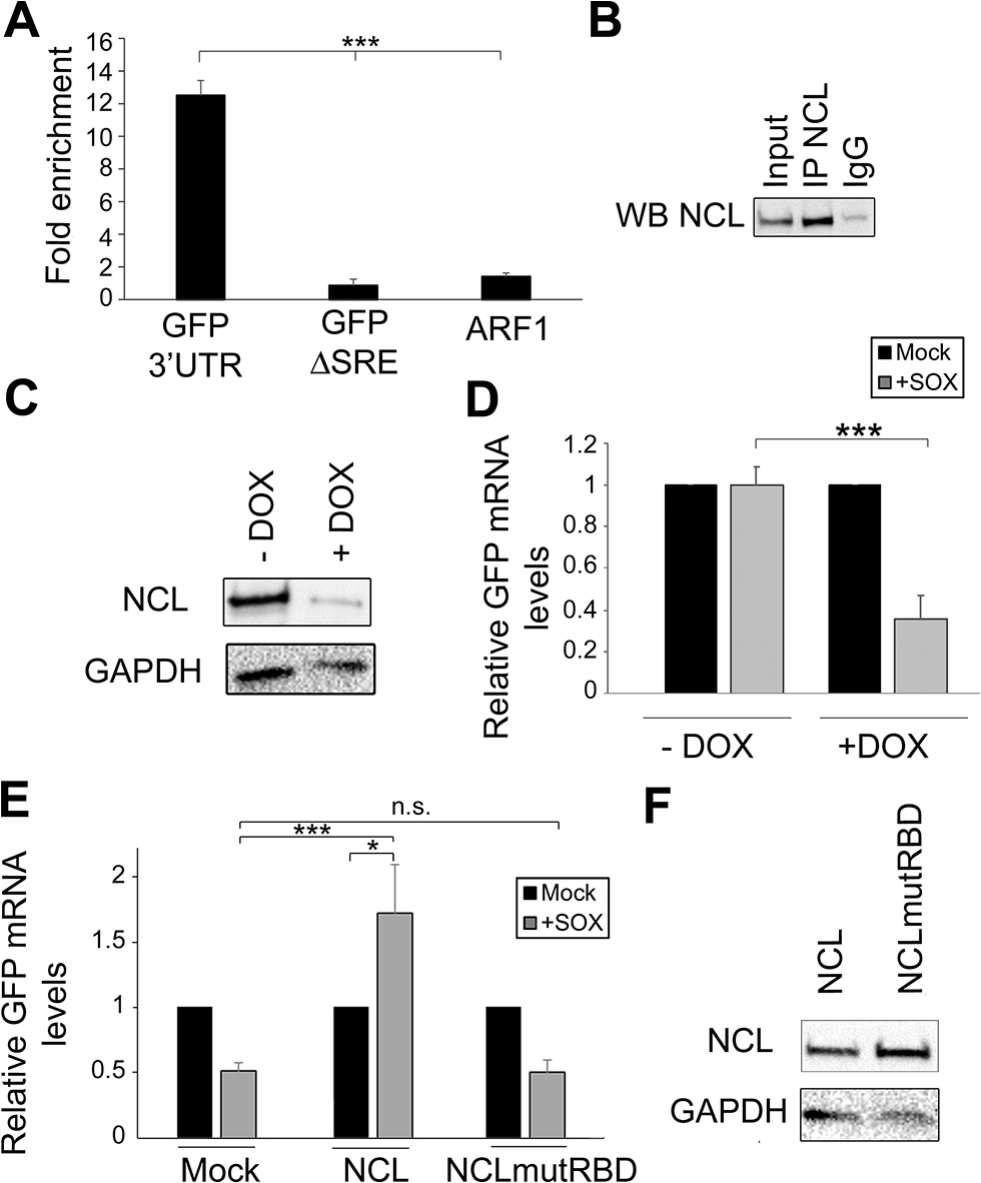
NCL binds the SRE in cells and contributes to IL-6 resistance. **(A)** 293T cells were transfected with the indicated GFP reporter, cross linked in 1% formaldehyde for 10 min, and lysed. Lysates were subjected to RNA immunoprecipitation (RIP) with anti-NCL (or mock IP with IgG) and the co-purifying mRNA was quantified by RT-qPCR. Bars represent the fold enrichment over mock IP. ARF1, an *a priori* NCL non-target, was included as a negative control. **(B)** Western blot showing the expression levels of NCL in the RIP input or post IP. **(C)** 293TΔNCL cells were treated with doxycycline (DOX) to induce NCL depletion, then lysates were Western blotted for NCL or GAPDH as loading control. **(D)** 293TΔNCL were incubated +/- DOX, then subsequently transfected with the GFP 3’ IL-6 plasmid alone or together with a SOX expression plasmid. 24h later GFP mRNA levels were quantified by RT-qPCR. **(E)** DOX-treated 293TΔNCL cells were co-transfected with GFP-3’ IL-6 and the indicated WT or mutant NCL expression plasmid +/- SOX. 24h later GFP mRNA levels were quantified by RT-qPCR. **(F)** Western blot showing the expression levels of NCL or GAPDH as loading control.

The strongest decrease in SRE-mediated protection was observed in cells depleted of NCL, and thus we decided to pursue this interaction in more detail. To confirm the interaction of NCL with the IL-6 SRE *in vivo*, we immunoprecipitated (IP) endogenous NCL from 293T cells transfected with either GFP-3’IL-6 or GFP-ΔSRE, and performed qRT-PCR to measure the level of co-precipitating RNA. We observed a ~10-fold enrichment of GFP-3’IL-6 over the mock (IgG) IP, but detected no enrichment of the GFP-ΔSRE construct or the negative control RNA ARF1 (an *a priori* non-NCL target) (**Fig 3A and 3B**). Thus, NCL exhibits an SRE-dependent interaction with the IL-6 3’ UTR *in vivo*.

NCL contains four RNA binding domains (RBD) that, when mutated, have been shown to compromise the ability of the protein to bind target RNA [21]. We therefore hypothesized that the RBD should be required for the ability of NCL to potentiate the protective effect of the SRE in complementation assays. To evaluate the importance of this domain in conferring protection from SOX, we first engineered 293T cells to stably express two doxycycline-inducible short hairpin (sh) RNAs targeting nucleolin (293TΔNCL). Doxycycline treatment of these cells resulted in an ~80% reduction of endogenous NCL protein (**Fig 3C**) and, in agreement with the NCL siRNA-based depletion data, rendered the GFP-3’IL-6 mRNA susceptible to SOX-induced degradation (**Fig 3D**). We first confirmed that the alterations in RNA abundance upon NCL depletion were due to changes in mRNA stability by measuring the half-life of GFP 3’ IL-6, SRE and ΔSRE in this cell line in the presence or absence of SOX (**S3 Fig**). We then constructed a mutant version of NCL in which key residues within RBD1 (F347/Y349) and RBD2 (I429/Y431) required for RNA binding were mutated to aspartic acid (NCLmutRBD) [21]. Although transfection of WT NCL into doxycycline-treated 293TΔNCL cells rescued protection of the GFP-3’IL-6 mRNA in the presence of SOX, no protective effect was conferred by transfection of the NCLmutRBD (**Fig 3E**). Ectopic expression of WT NCL not only rescued the protection phenotype, but also increased the basal levels of GFP expression. As we observed in S2 Fig, NCL depletion decreased GFP mRNA steady state levels, but ectopic expression of NCL rescued this decrease (**S4 Fig**), likely explaining the increase observed in Fig 3E. We confirmed by Western blotting that both proteins were expressed equivalently (**Fig 3F**). These observations demonstrate that NCL must bind to the SRE to confer protection against SOX.

### NCL is relocalized during lytic KSHV infection and protects IL-6 in the cytoplasm

**Fig 4:**
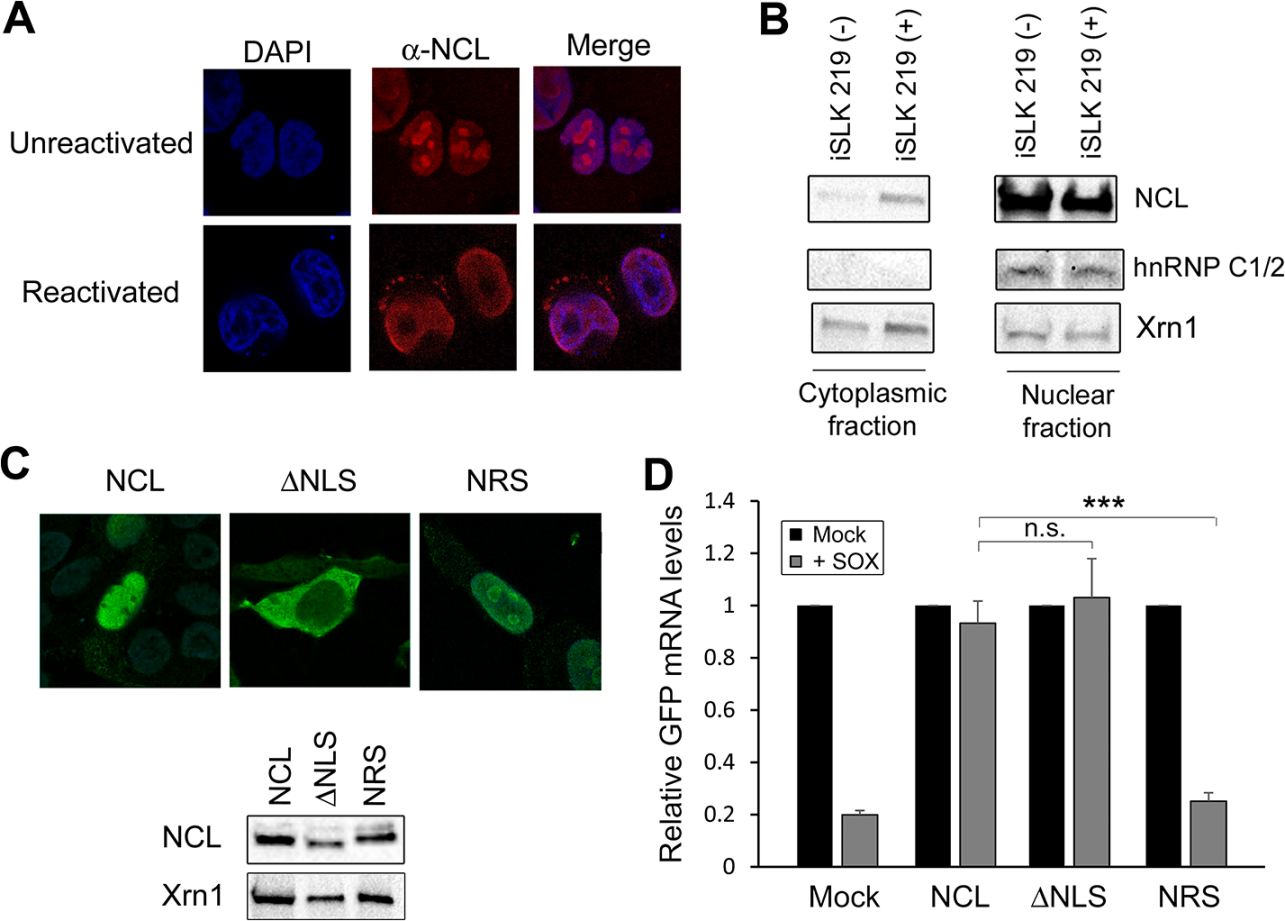
NCL is relocalized to the cytoplasm where it is required for IL-6 escape. **(A)** Unreactivated or DOX-treated KSHV-positive TREX-BCBL-1 cells were subjected to immunofluorescence assay using an anti-NCL antibody (red), and DAPI staining to identify nuclei (blue). **(B)** Unreactivated (-) or DOX-treated (+) KSHV-positive iSLK.219 cells were fractionated into nuclear and cytoplasmic fractions, and Western blotted for NCL, hnRNPC1/2 (nuclear fraction control) or Xrn1 (loading control; present in both compartments). **(C)** Expression of the NCL and the NCL ΔNLS or NRS mutants were visualized by immunofluorescence assay (*top*) and Western blot (*bottom*). **(D)** 293TΔNCL cells were treated with DOX and transfected with GFP-3’ IL-6 together with the indicated NCL expression plasmid in the presence or absence of SOX. 24h later GFP mRNA levels were quantified by RT-qPCR.

NCL is enriched in the nucleolus, but can also be present to a lesser extent in the nucleoplasm, cytoplasm, and at the plasma membrane [22,23]. Cleavage of mRNA by SOX takes place in the cytoplasm [24,25], and thus presumably sufficient cytoplasmic NCL must be present to ensure IL-6 protection during lytic KHSV infection. We monitored endogenous NCL localization in cells latently and lytically infected with KHSV by immunofluorescence assay (IFA) and by subcellular fractionation. First, we performed IFA for NCL in KSHV-positive TREX-BCBL-1 cells that were either latently infected or treated with doxycycline to induce lytic replication. In latently infected TREX-BCBL-1 cells, NCL expression was predominantly nucleolar, in agreement with previous reports [26] (**Fig 4A and S1 Video**). However, upon lytic reactivation, NCL localization shifted dramatically to the nucleoplasm and to punctate granules within the cytoplasm (**Fig 4A and S2 Video**).

We also used subcellular fractionation to monitor NCL localization in a second cell type, KSHV-positive iSLK.219 cells [27]. Similar to the TREX-BCBL-1 cells, iSLK.219 cells contain a doxycycline-inducible version of the major KSHV lytic transactivator RTA that enables lytic reactivation. In latently infected iSLK.219 cells, NCL remained almost exclusively nuclear (**Fig 4B**). However, in lytically reactivated iSLK.219 cells, a proportion of NCL was redistributed into the cytoplasm (**Fig 4B**). These results indicate that lytic KSHV infection induces re-localization of NCL, including into the cytoplasm where SOX-induced mRNA cleavage takes place.

NCL is a shuttling protein and contains in its N-terminal region a bipartite nuclear localization signal (NLS) [28]. To determine which population of NCL is important for SRE-mediated protection from SOX, we generated an NCL NLS mutant (NCLΔNLS) that was restricted to the cytoplasm, as well as a version of NCL fused to a nuclear retention signal (NRS-NCL) that was restricted to the nucleus (**Fig 4C**). We verified by Western blot (WB) that these constructs were expressed at similar levels (**Fig 4C**). It should be noted that although the intensity of the nuclear staining of WT NCL made it difficult to detect the cytoplasmic population by IFA, subcellular fractionation experiments confirmed that in 293T cells both endogenous and transfected WT NCL could be detected in both compartments (**S5 Fig**). We next evaluated the ability of each protein to rescue SRE-mediated escape from SOX degradation in the 293TΔNCL cell line. Both WT NCL and NCLΔNLS rescued levels of the GFP-3’IL-6 mRNA in the presence of SOX (**Fig 4D**). However, NRS-NCL was unable to rescue GFP3’IL-6 mRNA from SOX degradation (**Fig 4D**) Taken together, these results demonstrate that cytoplasmic NCL is involved in SRE-mediated protection.

Finally, we analyzed whether depletion of NCL from iSLK.219 cells by siRNA treatment impacted IL-6 mRNA levels and/or the lytic KSHV lifecycle. Indeed, NCL knockdown reduced the abundance of IL-6 mRNA during the KSHV lytic cycle compared to cells treated with control siRNAs (**S6A Fig**). This effect is not as robust as the effects of NCL depletion in the context of SOX transfection, likely because during infection KSHV has several other mechanisms to transcriptionally and post-transcriptionally increase IL-6 abundance [29,30]. We also detected a robust impairment of expression of KSHV late gene expression as measured by K8.1 levels, as well as a corresponding failure of the infected cells to produce progeny virions in supernatant transfer assays (**S6B and S6C Fig**). These results are supportive of a role for NCL in IL-6 protection in the context of KSHV infection, although NCL clearly plays additional crucial roles in the KSHV lifecycle.

### The SRE functions in a position-dependent manner and links NCL to the eIF4H translation factor

**Fig 5:**
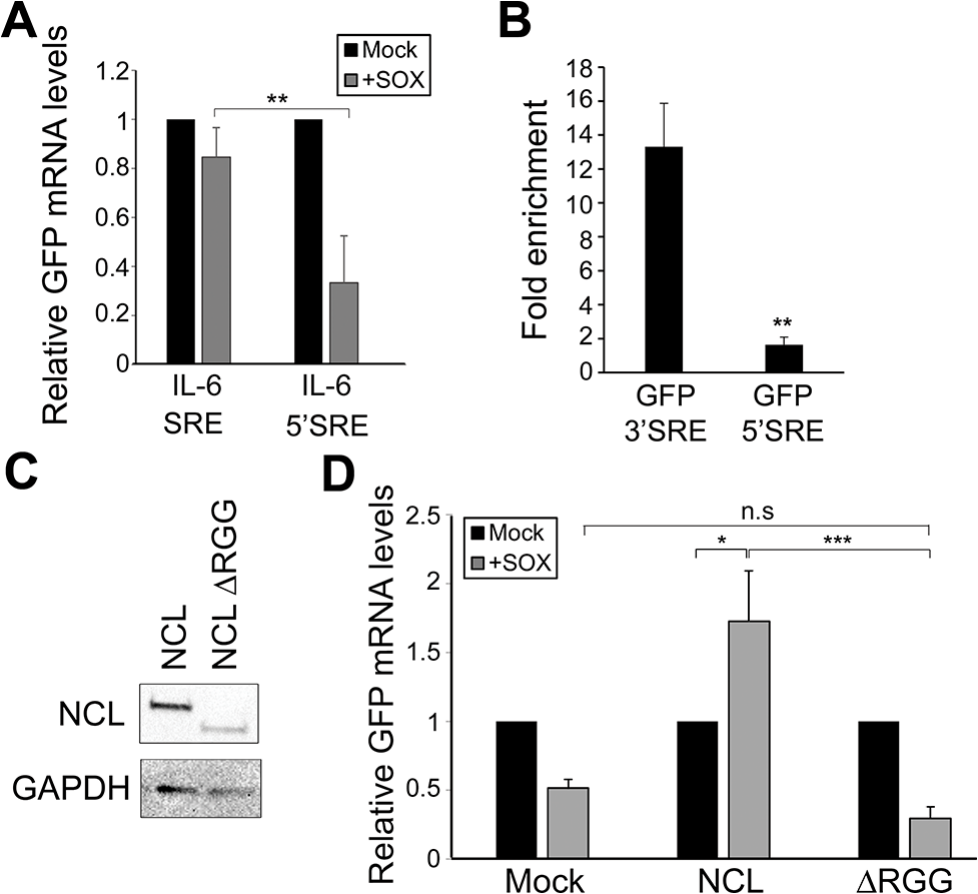
SRE-mediated protection is linked to its position and NCL protein-protein interactions. **(A)** 293T cells were co-transfected with the indicated GFP reporter plasmid in the presence or absence of SOX. After 24 h, GFP mRNA levels were quantified by RT-qPCR. **(B)** 293T cells were transfected with the indicated GFP reporter, cross linked in 1% formaldehyde for 10 min, and lysed. Lysates were subjected to RNA immunoprecipitation (RIP) with anti-NCL (or mock IP with IgG) and the co-purifying mRNA was quantified by RT-qPCR. Bars represent the fold enrichment over mock IP. **(C)** Anti-FLAG Western blot showing expression of NCL and NCLΔRGG. GAPDH was included as a loading control. **(D)** 293TΔNCL cells were treated with DOX and transfected with GFP-3’ IL-6 together with the indicated NCL expression plasmid in the presence or absence of SOX. 24h later GFP mRNA levels were quantified by RT-qPCR.

To determine whether the location of the SRE might impact protection, we tested the effect of moving the SRE from the 3’ UTR to the 5’ UTR on the GFP reporter (GFP-5’ SRE). Unlike the GFP-3’ SRE mRNA, the GFP-5’ SRE mRNA was degraded in SOX-expressing cells, indicating that SRE positioning is important (**Fig 5A**). This could be explained if ribosome scanning through the 5’ UTR disrupted NCL binding to the SRE, and/or if NCL positioning on an mRNA impacted its interactions with other mRNA-bound proteins to potentiate protection from SOX. We tested the first part of this hypothesis by measuring the efficiency with which NCL associated with the GFP-5’ SRE compared to GFP 3’ SRE. NCL displayed significantly reduced binding to the GFP-5’ SRE mRNA in RNA IPs, suggesting that the SRE RNP does not assemble efficiently if located in the 5’ UTR (**Fig 5B**).

**Fig 6:**
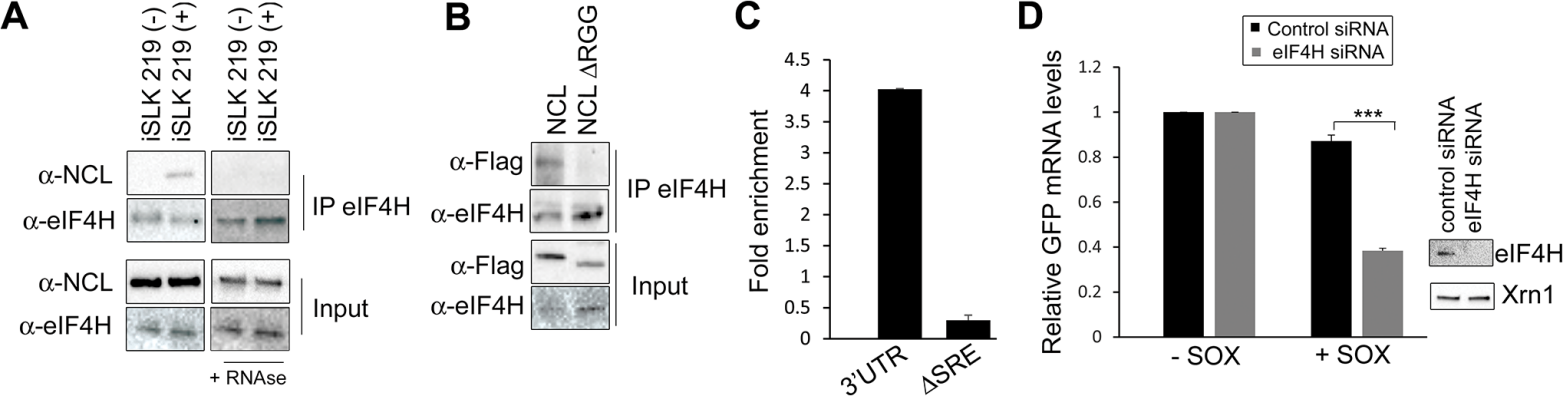
IL-6 escape is potentiated by an NCL-eIF4H interaction. **(A)** Lysates of latently infected (-) or DOX-reactivated (+) KSHV-positive iSLK.219 cells were subjected to IP with an anti-eIF4H antibody, then Western blotted (WB) for NCL or eIF4H. Where indicated, lysates were treated with RNAseA and RNAse T1. **(B)** 293T cells were transfected with the indicated FLAG-tagged NCL expression construct, subjected to IP with anti-eIF4H antibody, and Western blotted for FLAG-tagged proteins and eIF4H. **(C)** 293T cells were transfected with the indicated GFP reporter (3’UTR or ΔSRE) and FLAG-tagged NCLΔRGG, cross linked in 1% formaldehyde for 10 min, and lysed. Lysates were subjected to RNA immunoprecipitation (RIP) with anti-flag (or mock IP with IgG) and the co-purifying mRNA was quantified by RT-qPCR. Bars represent the fold enrichment over mock IP. **(D)** 293T cells were treated the indicated control or eIF4H-targeting siRNA. Cells were co-transfected 48h later with plasmids expressing the GFP IL-6 3’UTR reporter construct in the presence or absence of SOX. GFP mRNA levels were quantified by RT-qPCR (*left panel*), and eIF4H knockdown efficiency was monitored by Western blot (*right panels*). Xrn1 was included as a loading control.

We next pursued the idea that once recruited to the SRE, NCL-induced protection from SOX may involve its interaction with other cellular proteins. Previously described protein interactions of NCL largely occur through its C-terminal arginine-glycine repeat (RGG) region [23,31–35]. NCLΔRGG failed to protect the GFP-3’IL-6 mRNA from SOX in the doxycycline-treated 293TΔNCL cell line (**Fig 5C and 5D**), indicating a role for protein interactions in the SRE escape function of NCL. It should be noted that although the ΔRGG mutant is expressed at lower levels, increasing the amount transfected to produce levels matching those of WT did not rescue the protection phenotype (**S7 Fig**). Because the 5’ cap and 3’ poly(A) tail are defining mRNA features and positionally fixed, we hypothesized that interactions with one or more factors bound to these elements might impact the SRE-related function of NCL. We further reasoned that protein-protein interactions related to SOX resistance might be enhanced specifically during lytic KSHV infection, when NCL relocalization occurs. Using a targeted approach, we therefore searched for mRNA-associated factors that displayed selective or enhanced binding to NCL during lytic KSHV infection using co-immunoprecipitation (co-IP) assays. We found the cap-associated translation initiation factor eIF4H to selectively immunoprecipitate NCL from lytically but not latently infected iSLK219 cells (**Fig 6A**). This enrichment appeared specific to eIF4H, as we detected no differential interaction profile for NCL with additional mRNA cap- or tail-bound proteins including eIF4G, eIF4E, eIF4B and PABPC (**S8 Fig**). The interaction between NCL and eIF4H was disrupted when the lysates were treated with RNase (**Fig 6A**), in agreement with the idea that these proteins are not normally stably associated, but are brought together in the context of mRNA-bound NCL *via* a long-range interaction. Furthermore, the NCLΔRGG mutant failed to bind eIF4H in co-IP assays (**Fig 6B**), while still being able to bind specifically to a SRE containing reporter (**Fig 6C**), suggesting that the failure of this mutant to protect SRE-containing mRNAs from SOX may be due, at least in part, to its inability to bind eIF4H.

We reasoned that if the NCL-eIF4H interaction played a role in the escape of SRE-containing mRNAs from SOX cleavage, then depletion of eIF4H should decrease the efficiency of escape. Indeed, similar to our results with NCL, siRNA-mediated depletion of eIF4H rendered the GFP-3’IL-6 susceptible to degradation by SOX (**Fig 6D**). Depletion of eIF4H did not affect the expression of SOX, NCL, or XrnI, arguing against a generalized impediment of protein translation in these experiments (**S9 Fig**). This was not unexpected, given that an increasing number of translation factors previously thought to have generalized roles in translation, including the eIF4F complex, have instead been shown to be selectively required for only specific types of host mRNAs [36,37].

### The SRE also confers protection against the herpes simplex virus endonuclease vhs

**Fig 7:**
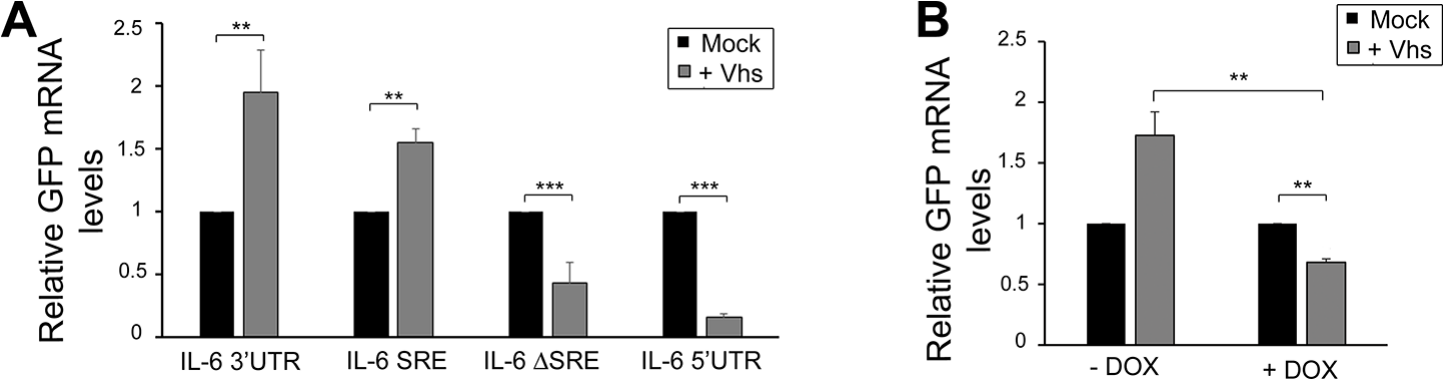
SRE-mediated protection mechanism expands beyond SOX induced decay. **(A)** 293T cells were transfected with GFP-3’ IL-6, GFP IL-6 SRE, GFP IL-6 ΔSRE or GFP-5’ IL-6 plasmids in the presence or absence of HSV-1 vhs. Cells were harvested 24h later and subjected to RT-qPCR to measure GFP levels. **(B)** 293TΔNCL cells were treated or not with DOX and transfected with GFP-3’ IL-6 in the presence or absence of vhs. 24h later GFP mRNA levels were quantified by RT-qPCR.

Viruses that promote widespread degradation of mRNA generally do so by encoding endonucleases or endonuclease-activating proteins [38–40]. We therefore explored the possibility that the IL-6 SRE might also confer protection against additional viral endonucleases. Herpes simplex virus (HSV) encodes an mRNA-targeting endonuclease (vhs) that, while not homologous to KSHV SOX, degrades most mRNAs during infection [41–44]. To test whether the SRE conferred protection against HSV-1 vhs, we measured by RT-qPCR the ability of vhs to degrade the GFP reporter mRNA fused to the IL-6 3’ UTR versus the control IL-6 5’ UTR. Intriguingly, the IL-6 3’ UTR as well as IL-6 SRE conferred complete protection from vhs, while the GFP mRNA containing the IL-6 5’UTR or ΔSRE was readily degraded (**Fig 7A**). To determine whether protection from vhs-mediated cleavage required NCL, we co-expressed vhs and GFP-3’IL-6 in the 293TΔNCL cell line. Upon Dox treatment to deplete NCL, GFP-3’IL-6 was no longer protected from vhs (**Fig 7B**). Thus, the SRE-containing IL-6 3’UTR can block mRNA cleavage by at least two non-homologous endonucleases via an NCL-dependent mechanism.

## Discussion

RNA degradation rates are heavily impacted by the cohort of proteins associated with each transcript, and here we define an RNP complex that inhibits viral endonuclease targeting. Unlike the majority of mRNAs in the cytoplasm that are degraded by the SOX endonuclease during lytic KSHV infection, the IL-6 mRNA is strongly induced and directly refractory to cleavage by SOX [8,17]. Although other mRNAs can also escape cleavage, IL-6 is the only mRNA known to escape *via* a dominant protective mechanism. We found that the 200 nt IL-6 protective element directs assembly of a large RNP complex, of which five components associated with the regulation of mRNA stability have now been shown to contribute to escape from SOX [18]. Notably, this escape element also functions to guard mRNAs against the HSV-1 vhs nuclease, despite the fact that vhs and SOX are unrelated and cleave mRNAs at distinct sites [11,45]. This key observation suggests that the underlying mechanism of escape does not involve a SOX-specific feature but instead must involve the general accessibility of the mRNA to these (and perhaps other) cytoplasmic endonucleases.

Although not homologous, SOX and vhs do share certain features in their RNA targeting strategies. Both proteins selectively cleave mRNA but not RNAs transcribed by RNA Polymerase I or III [44,46,47]. Furthermore, both proteins can target mRNAs prior to recruitment of the 40S ribosomal subunit, suggesting that ongoing translation of the target mRNA is not required for cleavage [46]. However, translation may nonetheless play some role in targeting, as vhs cleavage sites can be altered by mutating the target mRNA start codon or Kozak consensus context, and SOX can cleave mRNAs in polysomes [11,40,48]. While the factor(s) involved in recruiting SOX to its mRNA targets remain unknown, vhs recruitment involves interactions with the translation initiation factors eIF4H and eIF4AI/II [49,50]. Once brought to the mRNA, vhs tends to cleave in a cap-proximal manner in the 5’ UTR or near the start codon [48,51], whereas SOX requires a specific recognition sequence that can be located at sites far downstream from the cap [11,40,48]. The observation that SOX and vhs cleave mRNAs at distinct sites suggests that there must be differences in their mechanisms of targeting. In this regard, SRE-mediated protection could occur by blocking a factor required for both SOX and vhs recruitment to mRNAs. It is notable that eIF4H has been shown to bind vhs and help direct it to mRNAs [48,49,52,53]. However, unlike with vhs, no interaction between SOX and eIF4H have been reported in the literature, arguing against eIF4H accessibility as the feature underlying endonuclease escape for both vhs and SOX. The escape mechanism for these viral proteins is therefore expected to be different, although it is possible that an additional factor required for both SOX and vhs recruitment is occluded or displaced by the SRE RNP. Alternatively, the SRE may direct localization of the IL-6 mRNA into SOX- and vhs-inaccessible sites in the cytoplasm.

Although the fate of IL-6 during HSV-1 infection remains unknown, other specific mRNAs have been shown to escape degradation by vhs, some of which contain AU-rich elements in their 3’ UTR [54,55]. This has been studied most intensively for the IEX-1 mRNA, however, at present, reports differ as to what form of the IEX-1 mRNA is stabilized during infection and the precise role of vhs in altering IEX-1 mRNA decay [55–59]. Both GADD45β and the ARE-containing TTP mRNAs have also been shown to be directly refractory to vhs-mediated decay and are up-regulated at the protein level during HSV-1 infection [54,55,57]. Like IEX-1 and TTP, the IL-6 3’ UTR contains an ARE, which overlaps with the SRE [17]. While it is perhaps notable that the best-studied herpesviral escapees contain AREs, ARE-bearing mRNAs are not enriched in the overall pool of SOX escapees and the majority of ARE mRNAs are susceptible to degradation by SOX [13], arguing against this being the feature driving the IL-6 protective mechanism.

Among the proteins identified to selectively bind the SRE, NCL was the most potent modulator of escape. NCL has diverse roles in RNA biogenesis and has been previously shown to affect mRNA turnover [20]. Although the effects of NCL differ depending on the target transcript, it has been reported to interact with the 3′UTR of numerous mRNAs and enhance their stability. Known targets include amyloid precursor protein (APP), β-globin, Bcl-2, Bcl-xL, interleukin 2 (IL-2), and the growth arrest- and DNA damage-inducible 45 (GADD45A) [60–63]. NCL stabilization of mRNAs has also been linked to its ability to bind AREs [61,64,65]. One notable example is NCL-mediated stabilization of the GADD45A mRNA which, similar to IL-6, occurs via the binding of NCL at its 3’UTR [63]. GADD45A is one of the transcripts identified by RNAseq as being refractory to SOX cleavage [13] and to be up-regulated during HSV-1 infection [66]. Similar to what we observed during KSHV infection, stabilization of GADD45A is associated with the re-localization of NCL from dense nuclear foci to the nucleoplasm and cytoplasm upon arsenic-induced stress [63]. Thus, redistribution of NCL may contribute to stabilization of multiple stress-responsive mRNAs upon chemical or viral insults.

Although NCL clearly contributes to SRE function, the composition of the 200 nt SRE complicates the ability to make a direct and selective link between NCL and SOX escape. For example, a portion of the SRE contains AU-rich sequences, which are elements with established roles in the destabilization of many labile mRNAs [67]. Furthermore, the facts that NCL has been implicated in numerous aspects of mRNA biology and can impact the abundance of non-SRE mRNAs (such as GFP) highlight the broad effects this protein has in cells. Thus, it is possible that depletion of NCL has secondary effects on mRNA accumulation that indirectly influence the stability of IL-6 in SOX-expressing cells. Nonetheless, our observations that NCL binds specifically to the SRE and that this binding in the cytosol is required for protection against SOX suggest that at least some aspects of SRE-mediated escape from SOX are connected to the presence of NCL in the SRE-bound protein complex. In this regard, NCL-induced mRNA stabilization often involves its interaction with other RBPs, including HuR [63] and AUF1 [68], both of which are important for IL-6 escape from SOX degradation [18]. Given that NCL is a highly connected protein [23,31–35], it could act as a hub to assemble larger protein complexes, perhaps explaining its potent role in the escape mechanism. For example, it interacts with several of the identified SRE-binding proteins, including NPM1 [69,70] and STAU1 [71,72].

Here, we describe a novel interaction between NCL and the helicase accessory factor eIF4H. The fact that the NCL-eIF4H interaction selectively occurs during lytic but not latent KSHV infection suggests that this interaction is facilitated by NCL relocalization to the cytoplasm, although infection could also alter the translational requirements for eIF4H. The RNA-dependent nature of the NCL-eIF4H interaction indicates that these proteins associate in the context of mRNA-bound NCL, rather than freely in the cytoplasm, and demonstrates the importance of long-range interactions in mediating protection from SOX. Differential interactions between NCL and other translation-linked proteins were not observed, suggesting that NCL and eIF4H may selectively associate with specific mRNAs. In this regard, it is possible that eIF4H does not play a widespread role in translation but is instead recruited to a subset of mRNAs including IL-6. While not yet explored for eIF4H, it has recently been shown that the cap binding complex eIF4F is preferentially required for the translation of mRNAs that contain 5’ pyrimidine-rich elements [36,37]. Furthermore, eIF4H has a closely related homolog in mammalian cells, eIF4B, which might play a role redundant to that of eIF4H on other mRNAs [73,74]. It will be of interest to determine whether additional NCL-bound mRNAs recruit eIF4H and, additionally, whether other NCL and eIF4H-bound mRNAs escape SOX and vhs. Furthermore, it would be interesting to explore whether KSHV infection favors differential expression of the translation initiation complex components and whether this influences viral gene expression and/or escape from viral induced host shutoff.

Cytoplasmic NCL has been shown to be co-opted by a diverse set of viruses, including to help mediate the human respiratory syncytial virus entry, HIV gag complex assembly, and poliovirus virion formation [75–78]. NCL also plays a positive role in HSV-1 infection [75], where, similar to KSHV infection, it is relocalized to the nucleoplasm and cytoplasm [79,80]. Thus, although NCL is directly involved in mediating protection of IL-6, it is likely that its cytoplasmic relocalization during KSHV reactivation plays additional roles in the viral lifecycle, as its depletion also causes strong defects in K8.1 late gene expression and virion production. At present it is difficult to distinguish its role in IL-6 mRNA accumulation during KSHV infection from its additional crucial roles in the viral life cycle. NCL localization has been linked to its phosphorylation state [81,82]. Thus, one possibility is that NCL is phosphorylated by one of the herpesviral protein kinases, although infection may also activate NCL-targeting cellular kinase cascades. Future studies are anticipated to reveal whether the cytoplasmic population of NCL is posttranslationally modified during infection, and whether this facilitates its interactions that form the basis for SRE-mediated protection.

## Materials and Methods

### Cells and transfections

The KSHV-positive B cell line bearing a doxycycline-inducible version of the major lytic transactivator RTA (TREX-BCBL-1) [83] was maintained in RPMI medium (Invitrogen) supplemented with 10% fetal bovine serum (FBS; Invitrogen), 200 μM L-glutamine (Invitrogen), 100 U/ml penicillin/streptomycin (Invitrogen), and 50 μg/ml hygromycin B (Omega Scientific). Lytic reactivation was induced by treatment with 20 ng/ml 2-O-tetradecanoylphorbol-13-acetate (TPA; Sigma), 1 μg/ml doxycycline (BD Biosciences), and 500 ng/ml ionomycin (Fisher Scientific) for 48h. 293T cells (ATCC) were grown in DMEM (Invitrogen) supplemented with 10% FBS. The KHSV-infected renal carcinoma cell line iSLK.219 bearing doxycycline-inducible RTA were grown in DMEM supplemented with 10% FBS [84]. KSHV lytic reactivation of the iSLK.219 cells was induced by the addition of 0.2 μg/ml doxycycline (BD Biosciences) and 110 μg/ml sodium butyrate for 48 h. For supernatant transfer experiments, the supernatant of iSLK.219 cells reactivated or not was collected after 48h, filtered through a. 45uM filter and spinfected onto fresh 293T for 1h at 1500rpm. Cells were then fixed and mounted onto slides to visualize with a confocal microscopy on a Zeiss LSM 710 AxioObserver microscope. 293TΔNCL were generated by lentiviral transduction. Briefly, psPAX2 and pMD2.G lentiviral plasmids were co-transfected with pTRIPZ plasmids encoding Doxyclyclin inducible shRNAs targeting NCL (V2THS_36645 and V2THS_36643 from Open Biosystems, kindly provided by Chih-Wen Peng at Tzu-Chi University). Supernatant containing viral particles was collected 48h later, filtered, complemented with 8 μg/mL polybrene and centrifuged onto target 293T cells.

For DNA transfections, cells were plated and transfected after 24h when 70% confluent using linear PEI (polyethylenimine). For small interfering RNA (siRNA) transfections, 293T cells were reverse transfected in 12-well plates by INTERFERin (Polyplus-Transfection) with 10 μM of siRNAs. siRNAs were obtained from IDT as DsiRNA and sequences are as described in **S3 Table**. 48h following siRNA transfection, the cells subjected to DNA transfection as indicated.

For time course experiments, half-live were measured by transfecting 293T or 293TΔNCL cells with the indicated plasmids in 6-well plates. The cultures were split after 6 h into 12-well plates and 12 h later treated with 5 μg/mL Actinomycin D (ActD) for the indicated times. The extracted RNAs were subjected to qPCR analysis and GFP mRNA levels were normalized to the level of 18S rRNA.

### Plasmids

The full-length IL-6 cDNA in pCMV-SPORT6.1 was obtained from Invitrogen. Sequence numbering for IL-6 refers to *Homo sapiens* interleukin 6 (interferon, beta 2), mRNA (GenBank accession number BC015511.1). The GFP-IL-6 3’UTR and GFP-IL-6 SRE fusion constructs were described previously [18], and the GFP-IL-6 3’UTR ΔSRE construct was obtained by overlap PCR into the pcDNA3.1 IL-6 3’UTR plasmid cut with BlpI and XbaI with the following primers (primers sequences are described in **S4 Table**); IL-6 ΔSRE PCR1 and PCR2, forward and reverse. The Csy4 recognition motif was fused to the SRE or IL-6 nucleotide sequence 251–450 by PCR with the Csy4 primers (**S4 Table**) and cloned into the KpnI and XhoI sites of pcDNA3.1.

NCL was obtained from 293T total cDNA and cloned into the Gateway entry vector pDON207 (Invitrogen) using the following primers (**S4 Table**): NCL-Forward and Reverse. It was then transferred into the gateway-compatible destination vector pCiNeo-3xFlag to generate Flag-NCL fusions. For the NCL ΔRGG mutant, the same forward primer was used, but with ΔRGG Reverse primer was. Other mutations were introduced with the Quickchange site directed mutagenesis protocol (Agilent) using the following primers: NCL ΔNLS; NCLmutRBD mutant was generated in a two-step process to introduce mutations both in the RNA binding domains 1 and 2 as described in [21]: RBD1 mutating F347 and Y349 into D in NCL RBD1 domain; to generate the final NCLmutRBD, this RBD1 mutant was further mutated at residues I429 and Y431 into D in the RBD2 domain. The final NCLmutRBD mutant thus contains 4 mutations.

### Csy4 pull down and mass spectrometry

Csy4 H29A/S50C was expressed and purified using the same protocol as wild-type Csy4 (generously provided by R. Haurwitz, H.Y. Lee, and J. Doudna) [19,85]. Plasmids expressing the Csy4 RNA binding motif fused to segments of IL-6 were *in vitro* transcribed using the T7 Maxiscript kit (Ambion). Transcribed RNA (20 μg) was mixed with purified recombinant Csy4 protein (200 pmol) and magnetic beads for 2h in lysis buffer [10 mM HEPES (pH 8.0), 3 mM MgCl2, 5% glycerol, 1 mM dithiothreitol (DTT), 150 mM NaCl, 0.1% octyl β-d-glucopyranoside, 10 mM imidazole, 1× protease inhibitor]. Lysate from TREX-BCBL1 or 293T cells (1 mg) was then added to the beads for 2h, whereupon the beads were washed 7 times with lysis buffer containing 150 to 300 mM NaCl. RNA and its associated cellular proteins were released from the Csy4-bound beads by the addition of 500 mM imidazole for 2h to activate the cleavage activity of Csy4. Eluates were processed, trypsin digested, and concentrated for LC-MS/MS. Digested peptide mixtures were analyzed by LC-MS/MS on a Thermo Scientific Velos Pro ion trap mass spectrometry system equipped with a Proxeon Easy nLC II high pressure liquid chromatography and autosampler system.

### Protein identification using reversed-phase liquid chromatography electrospray tandem mass spectrometry (LC-MS/MS)

Specific protein bands were excised from a gel and subjected to in-gel tryptic digestion. The gel bands were reduced with 10 mM dithiothreitol (Sigma-Aldrich) at 56°C for 1 hour, followed by alkylation with 55 mM iodoacetamide (Sigma) at room temperature in dark for 45 minutes. The samples were then incubated overnight with 100ng trypsin (Promega) at 37°C. The peptides formed from the digestion were extracted using 50% acetronitrile and 5% formic acid, and then re-suspended in 10 μl of 0.1% formic acid in water and analyzed by on-line LC-MS/MS technique. The LC separation was performed using a NanoAcquity UPLC system (Waters) while the MS/MS analysis was performed using a LTQ Orbitrap XL mass spectrometer (Thermo Scientific). During the LC separation step, 0.1% formic acid in water was used as the mobile phase A and 0.1% formic acid in acetonitrile was employed as the mobile phase B. Following the initial equilibration of the column in 98% A /2% B, 5 μL of the sample was injected. A linear gradient was started with 2% B and increased to 25% B in 33 mins followed by an increase to 60% B in the next 12 mins at a flow rate of 400 nL/min. The subsequent MS analysis was performed using a top six data-dependent acquisition. The sequence includes one survey scan in the FT mode in the Orbitrap with mass resolution of 30,000 followed by six CID scans in LTQ, focusing on the first six most intense peptide ion signals whose m/z values were not in the dynamically updated exclusion list and their intensities were over a threshold of 1000 counts. The analytical peak lists were generated from the raw data using an in-house software, PAVA [86]. The MS/MS data were searched against the UniProt database using an in-house search engine Protein Prospector (http://prospector.ucsf.edu/prospector/mshome.htm).

### RT-qPCR

Total RNA was harvested using Zymo RNA extraction columns following the manufacture's manual. cDNAs were synthesized from 1 μg of total RNA using AMV reverse transcriptase (Promega), and used directly for quantitative PCR (qPCR) analysis with the DyNAmo ColorFlash SYBR green qPCR kit (Thermo Scientific). Signals obtained by qPCR were normalized to 18S.

### Immunoprecipitation

Cells were lysed in low-salt lysis buffer [NaCl 150mM, NP-40 0.5%, Tris pH8 50mM, DTT 1mM, protease inhibitor cocktail] and protein concentrations were determined by Bradford assay. Equivalent quantities of each sample were incubated overnight with the indicated antibody, and then with G-coupled magnetic beads (Life technologies) for 1h. Where indicated, specific beads coupled to antibodies were used (M2 anti-flag beads; Sigma). Beads were washed extensively with lysis buffer. Samples were resuspended in Western blot loading buffer before resolution by SDS-PAGE. Where indicated, RNAse A and T1 were added to the lysates.

### Western blotting

Cell lysates were prepared in lysis buffer and quantified by Bradford assay. Equivalent amounts of each sample were resolved by SDS-PAGE and Western blotted with the following antibodies: Rabbit anti-NCL (Abcam), Mouse anti-NCL (Santa Cruz), Rabbit anti-eIF4H (Cell signaling). Rabbit anti-Flag (Sigma), Mouse anti hnRNPC1/C2 (Abcam), Rabbit anti-H3 (Cell Signaling), Rabbit anti-Xrn1 (Sigma), Mouse anti-Strep (Qiagen). Primary antibodies were followed by HRP-conjugated goat anti-mouse or goat anti-rabbit secondary antibodies (Southern Biotechnology, 1:5000).

### Immunofluorescence assays

293T or TREX-BCBL1 cells were grown on coverslips, and fixed in 4% formaldehyde for 20 min at room temperature. Cells were then permeabilized in 1% Triton-X-100 and 0.1% sodium citrate in PBS for 10 min, saturated in BSA for 30 min and incubated with the indicated antibodies. After 1h, coverslips were washed in PBS and incubated with AlexaFluor594 or AlexaFluor488 secondary antibodies at 1:1500 (Invitrogen). Coverslips were washed again in PBS and mounted in DAPI-containing Vectashield mounting medium (VectorLabs) to stain cell nuclei before visualization by confocal microscopy on a Zeiss LSM 710 AxioObserver microscope.

### Statistical analysis

All results are expressed as means ± S.E.M. of experiments independently repeated at least three times. Unpaired Student's t test was used to evaluate the statistical difference between samples. Significance was evaluated with pValues as follows: * p<0.1; ** p<0.05; *** p<0.01.

## Supporting Information

S1 Fig IL-6 SRE is sufficient to prevent degradation from SOX. 293T cells were cotransfected with plasmids expressing GFP 3’ IL-6, SRE or ΔSRE in the presence or absence of SOX. Eighteen hours post-transfection, cells were treated with actinomycin D for the indicated times and the levels of GFP relative to 18S at each time point were calculated after qPCR.
(PDF)

S2 Fig Effect of siRNA treatment on GFP mRNA steady state levels. 293T cells were transfected with a control siRNA or siRNAs targeting the 5 SRE-binding proteins with the strongest effect on IL-6 escape. After 48h, total RNA was collected and GFP mRNA levels were quantified by RT-qPCR.
(PDF)

S3 Fig NCL depletion affects RNA degradation rates. 293TΔNCL cells treated with Dox to deplete NCL were cotransfected with plasmids expressing GFP 3’ IL-6, SRE or ΔSRE in the presence or absence of SOX. Eighteen hours post-transfection, cells were treated with actinomycin D for the indicated times and the levels of GFP relative to 18S at each time point were calculated after qPCR. T_1/2_ measurements were derived from the exponential fit equations.
(PDF)

S4 Fig NCL ectopic expression rescues over basic levels the steady state of GFP mRNA. 293T cells were transfected with a control siRNA or siRNA targeting NCL (siNCL). After 48h, cells were further transfected with WT NCL. 24h later, total RNA was collected and GFP mRNA levels were quantified by RT-qPCR.
(PDF)

S5 Fig Endogenous NCL is expressed both in the nucleus and in the cytoplasm of 293T cells. 293T cells were fractionated and Western blotted using antibodies against NCL and H3 (as a nuclear fraction control).
(PDF)

S6 Fig Effect of NCL knock down during iSLK.219 lytic reactivation. iSLK.219 were treated with a control siRNA or a siRNA targeting NCL (siNCL). After 48h, cells were reactivated (+) or not (-) for 48h and used in three different assays: RT-qPCR to measure endogenous IL-6 mRNA levels (**A**); Western Blot to control the protein expression levels of K8.1 (late gene), NCL and Xrn1 as a control (**B**); and in a supernatant transfer assay as a proxy for virion production (**C**).
(PDF)

S7 Fig Increased amounts of NCL ΔRGG are still not sufficient to rescue protection. 293TΔNCL cells were treated with DOX and transfected with GFP-3’ IL-6 together with the WT NCL and the ΔRGG expression plasmid in amounts resulting in similar protein expression levels in the presence or absence of SOX. 24h later GFP mRNA levels were quantified by RT-qPCR and protein levels were assessed by WB using an anti-Flag antibody.
(PDF)

S8 Fig Interaction of NCL with mRNA binding proteins. Lysates of latent (-) or DOX-reactivated (+) KSHV-positive iSLK.219 were subjected to immunoprecipitation (IP) and Western blotting with the indicated antibodies.
(PDF)

S9 Fig Effect of eIF4H knock down on protein levels. 293T cells were transfected with a control siRNA or a siRNA targeting eIF4H (sieIF4H). After 48h, cells were transfected with SOX. 24h later cells were lysed and Western blotted to monitor the levels of Xrn1, SOX, NCL and eIF4H.
(PDF)

S1 Video NCL is predominantly nucleolar in unreactivated TREX-BCBL-1 cells. 3D reconstruction of an unreactivated TREX-BCBL-1 cell showing endogenous NCL (red) and DAPI-stained nuclei (blue) obtained using Z-stack images with Zen software.
(AVI)

S2 Video NCL is relocalized in lytically reactivated TREX-BCBL-1 cells. 3D reconstruction of a reactivated TREX-BCBL-1 cell showing endogenous NCL (red) and DAPI-stained nuclei (blue) obtained using Z-stack images with Zen software.
(AVI)

S1 Table Complete list of proteins identified through mass spectrometry. All proteins identified by MS after Csy4 pull down, including accession number and peptide count. All proteins listed were identified in either TREX-BCBL-1 or 293T and using either IL-6 SRE or a IL-6 nt 251–450.
(XLSX)

S2 Table Go term analysis of MS results shows 7 clusters. High confidence SRE interacting proteins were subjected to GO term analysis using DAVID bioinformatics database. This table lists the 7 clusters, the GO terms and the proteins involved in the clusters.
(XLSX)

S3 Table siRNA target sequences. (XLSX)

S4 Table Sequences of primers used for cloning. (XLSX)
