# Supplementary figures and images for: A Ribonucleoprotein Complex Protects the Interleukin-6 mRNA from Degradation by Distinct Herpesviral Endonucleases
Source: PLoS Pathog. 2015 May 12;11(5):e1004899. doi: 10.1371/journal.ppat.1004899 (PMC4428876; doi:10.1371/journal.ppat.1004899)

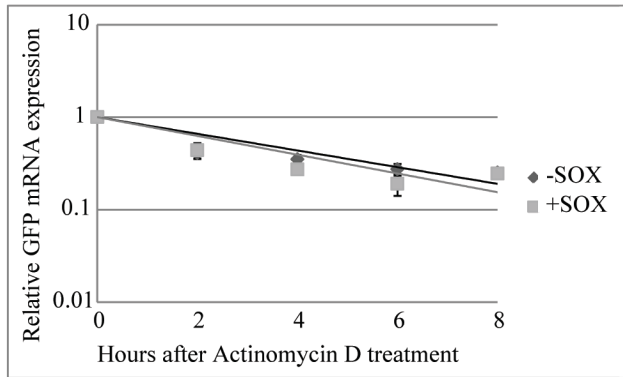

GFP IL-6 3'UTR

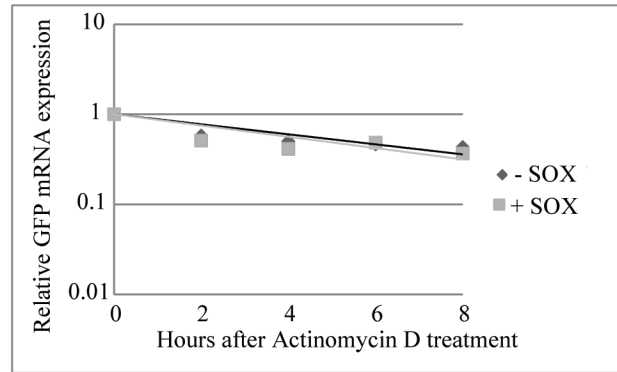

GFP IL-6 SRE

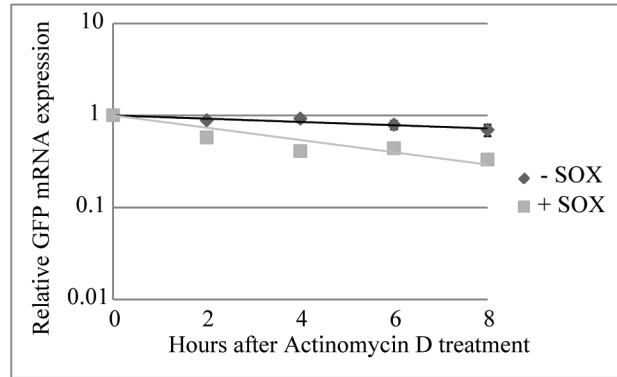

GFP IL-6 ΔSRE

Supplement: S1 Fig — 293T cells were cotransfected with plasmids expressing GFP 3’ IL-6, SRE or ΔSRE in the presence or absence of SOX. Eighteen hours post-transfection, cells were treated with actinomycin D for the indicated times and the levels of GFP relative to 18S at each time point were calculated after qPCR. (PDF) [file ppat.1004899.s001.pdf]

Relative GFP mRNA levels

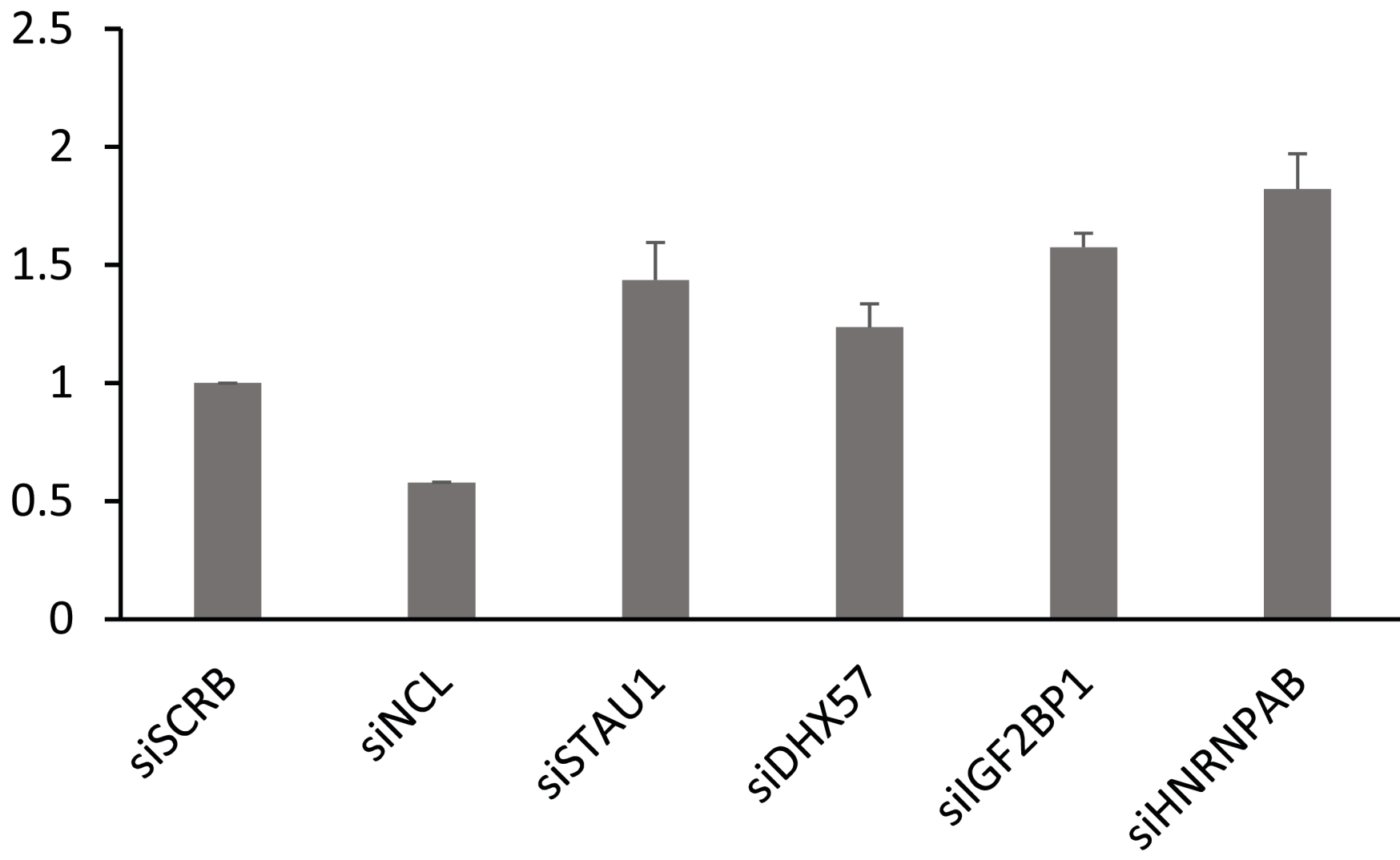

Supplement: S2 Fig — 293T cells were transfected with a control siRNA or siRNAs targeting the 5 SRE-binding proteins with the strongest effect on IL-6 escape. After 48h, total RNA was collected and GFP mRNA levels were quantified by RT-qPCR. (PDF) [file ppat.1004899.s002.pdf]

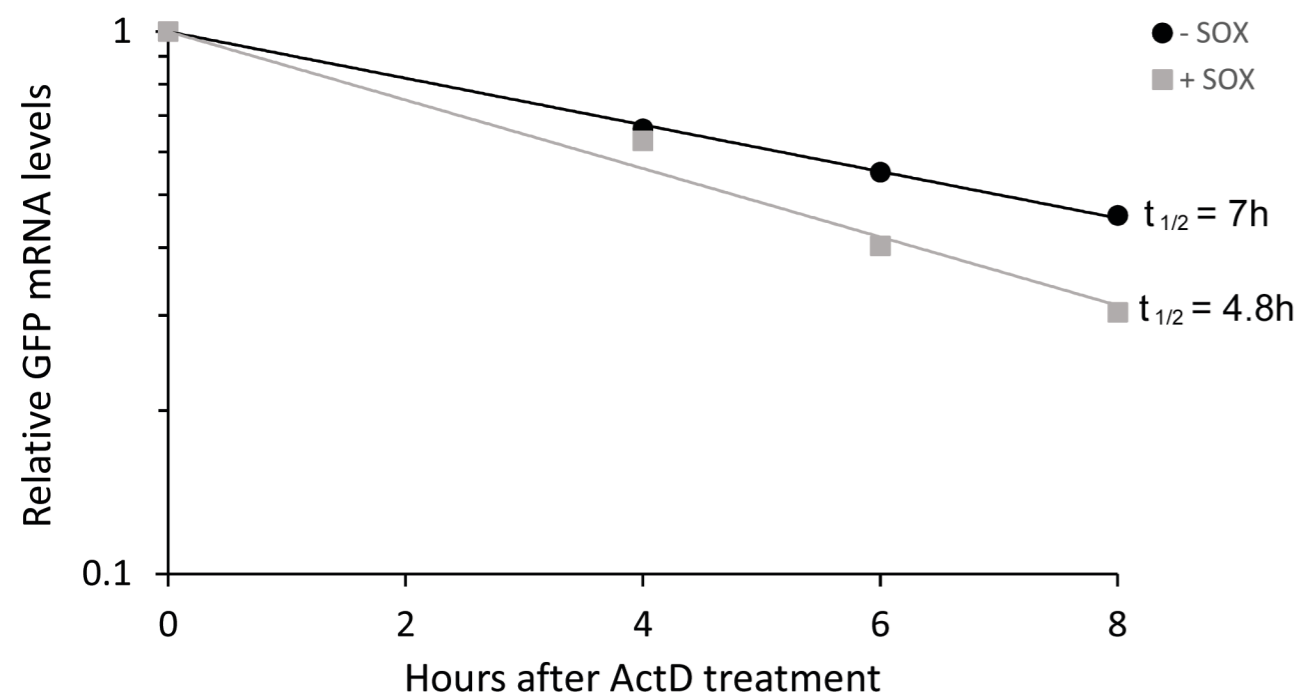

GFP 3'UTR

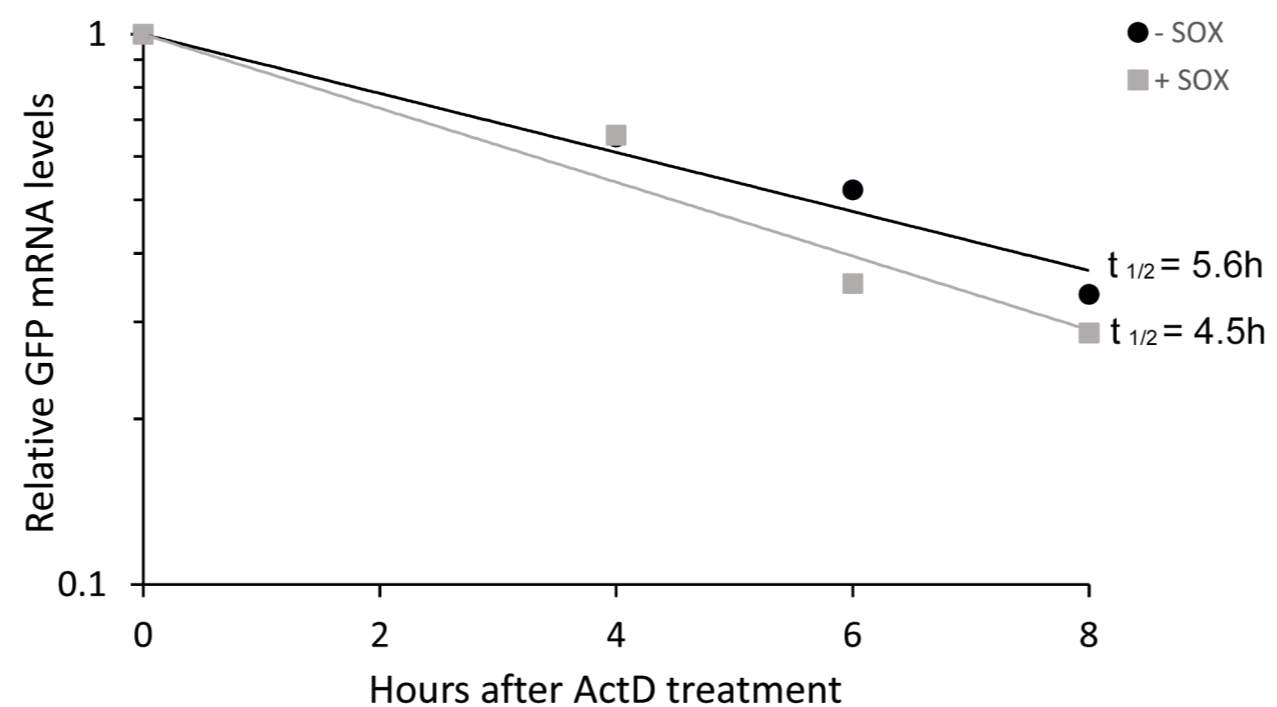

GFP SRE

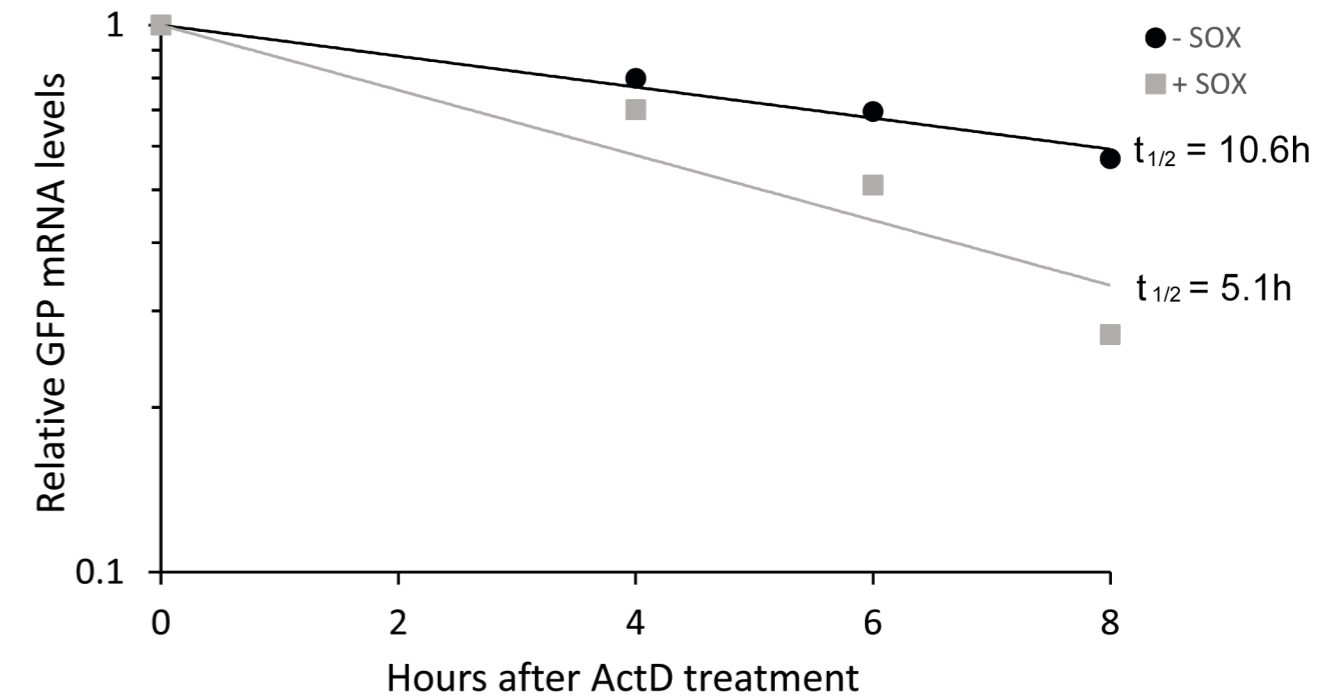

GFP  $\Delta$ SRE

Supplement: S3 Fig — 293TΔNCL cells treated with Dox to deplete NCL were cotransfected with plasmids expressing GFP 3’ IL-6, SRE or ΔSRE in the presence or absence of SOX. Eighteen hours post-transfection, cells were treated with actinomycin D for the indicated times and the levels of GFP relative to 18S at each time point were calculated after qPCR. T1/2 measurements were derived from the exponential fit equations. (PDF) [file ppat.1004899.s003.pdf]

Relative GFP mRNA levels

3  
2.5  
2  
1.5  
1  
0.5  
0

siSCRB

siNCL

siNCL + NCL

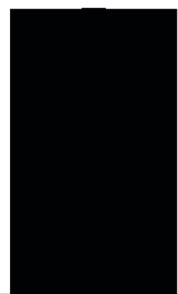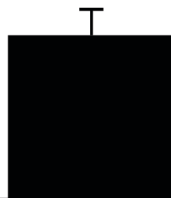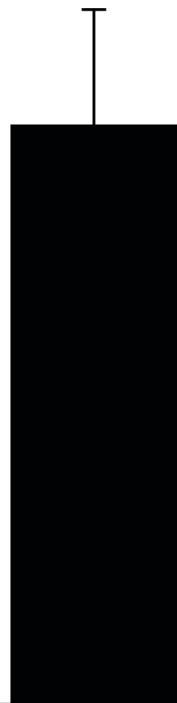

Supplement: S4 Fig — 293T cells were transfected with a control siRNA or siRNA targeting NCL (siNCL). After 48h, cells were further transfected with WT NCL. 24h later, total RNA was collected and GFP mRNA levels were quantified by RT-qPCR. (PDF) [file ppat.1004899.s004.pdf]

Nuclear Fraction  
Cytoplasmic Fraction

293T

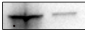

NCL

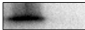

H3

Supplement: S5 Fig — 293T cells were fractionated and Western blotted using antibodies against NCL and H3 (as a nuclear fraction control). (PDF) [file ppat.1004899.s005.pdf]

**A.**

Relative IL-6 mRNA levels

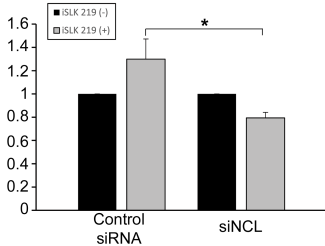**B.**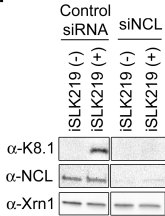**C.**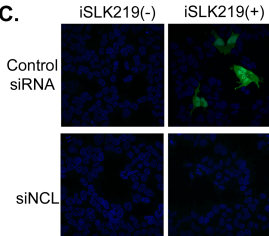

Supplement: S6 Fig — iSLK.219 were treated with a control siRNA or a siRNA targeting NCL (siNCL). After 48h, cells were reactivated (+) or not (-) for 48h and used in three different assays: RT-qPCR to measure endogenous IL-6 mRNA levels (A); Western Blot to control the protein expression levels of K8.1 (late gene), NCL and Xrn1 as a control (B); and in a supernatant transfer assay as a proxy for virion production (C). (PDF) [file ppat.1004899.s006.pdf]

Relative GFP mRNA levels

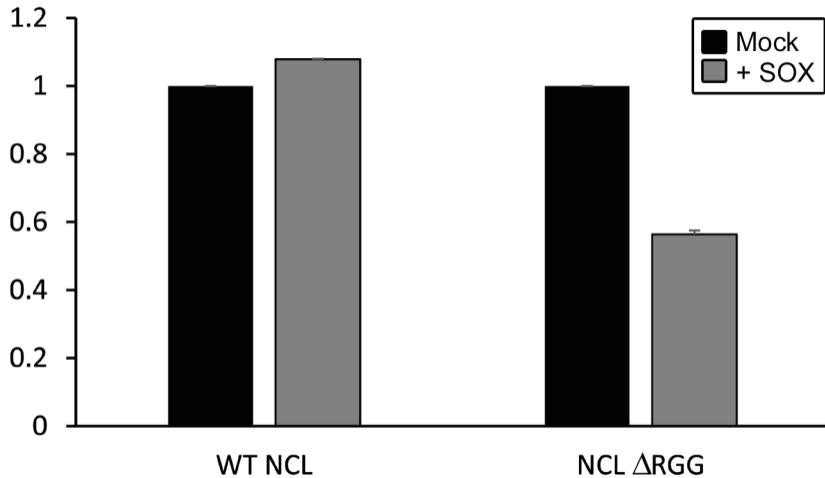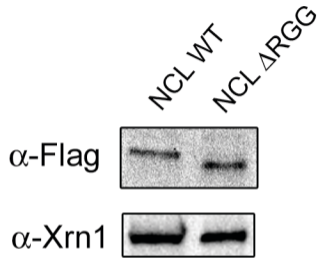

Supplement: S7 Fig — 293TΔNCL cells were treated with DOX and transfected with GFP-3’ IL-6 together with the WT NCL and the ΔRGG expression plasmid in amounts resulting in similar protein expression levels in the presence or absence of SOX. 24h later GFP mRNA levels were quantified by RT-qPCR and protein levels were assessed by WB using an anti-Flag antibody. (PDF) [file ppat.1004899.s007.pdf]

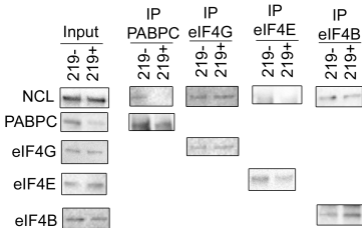

Supplement: S8 Fig — Lysates of latent (-) or DOX-reactivated (+) KSHV-positive iSLK.219 were subjected to immunoprecipitation (IP) and Western blotting with the indicated antibodies. (PDF) [file ppat.1004899.s008.pdf]

control siRNA

eIF4H siRNA

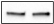

Xmi1

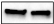

NCL

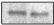

SOX

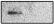

eIF4H

Supplement: S9 Fig — 293T cells were transfected with a control siRNA or a siRNA targeting eIF4H (sieIF4H). After 48h, cells were transfected with SOX. 24h later cells were lysed and Western blotted to monitor the levels of Xrn1, SOX, NCL and eIF4H. (PDF) [file ppat.1004899.s009.pdf]
